# Genome-Wide Analysis of the *LEA* Gene Family in Pineapple (*Ananas comosus* L.) Reveals Its Potential Roles in Cold Stress Response and Reproductive Development

**DOI:** 10.3390/biology14121655

**Published:** 2025-11-24

**Authors:** Zhimin Hou, Xinkai Cai, Denghang Wu, Haichao Gong, Jing Wang, Yinan Zhang, Qinglong Yao, Lulu Wang, Yuqin Liang, Yangmei Zhang, Yuan Qin, Xiaomei Wang, Ping Zheng

**Affiliations:** 1 Fujian Provincial Key Laboratory of Haixia Applied Plant Systems Biology, Haixia Institute of Science and Technology and College of Life Sciences, Fujian Agriculture and Forestry University, Fuzhou 350002, China; summer20200505@163.com (Z.H.); caixinkai2003@163.com (X.C.); wudenghang242412@163.com (D.W.); gonghaichao2025@163.com (H.G.); wangjing051402@163.com (J.W.); m15859928860@163.com (Y.Z.); yql_0418@163.com (Q.Y.); luluwanghn@163.com (L.W.); yuanqin@fafu.edu.cn (Y.Q.); 2 Rice Research Institute, Fujian Academy of Agricultural Sciences, Fuzhou 350018, China; 3 Horticulture Research Institute, Guangxi Academy of Agricultural Sciences, Nanning Investigation Station of South Subtropical Fruit Trees, Ministry of Agriculture, Nanning 530004, China; 4 Xiamen Botanical Garden, Xiamen 361000, China; liangyuqin5@sina.com; 5 Yunnan Institute of Tropical Crops, Jinghong City, Xishuangbanna 666100, China; yangmeiz66@163.com

**Keywords:** pineapple, late embryogenesis abundant (LEA) proteins, expression pattern, cold tolerance, reproductive development

## Abstract

Late Embryogenesis Abundant (LEA) proteins play vital roles in plant responses to abiotic stress and developmental regulation. Pineapple (*Ananas comosus* L.) is a major tropical fruit crop with high economic value, but its production is often threatened by cold stress, particularly in regions at the northern margin of its cultivation. Despite the recognized importance of LEA proteins in stress adaptation, their genomic landscape and functional characteristics in pineapple remain largely unexplored. In this study, 37 *AcLEA* genes were identified in the pineapple (*Ananas comosus* L.) genome and classified into six subfamilies, with *LEA_2* being the largest. Most AcLEA proteins were predicted to be hydrophilic, thermally stable, and intrinsically disordered, consistent with typical LEA protein characteristics. Phylogenetic and collinearity analyses revealed species-specific expansion patterns, primarily driven by segmental duplication events. Most duplicated gene pairs shared similar exon–intron structures, motif compositions, and expression profiles, although several displayed signs of functional divergence based on distinct expression patterns, Ka/Ks ratios > 1, and motif differences. Promoter *cis*-element, transcription factor, and miRNA network predictions indicated that *AcLEA* genes are widely involved in stress responses as well as growth and development. Expression profiling showed that many *AcLEA* genes including *AcLEA32*, *AcLEA7*, *AcLEA9*, *AcLEA30*, *AcLEA29*, *AcLEA33*, and *AcLEA18* were significantly upregulated under cold stress and declined upon stress removal, indicating a potential role in cold tolerance. Some *AcLEA* genes, such as *AcLEA32* and *AcLEA33*, showed faster and stronger induction under cold stress in the cold-tolerant cultivar “Comte de Paris” (BL) compared to the sensitive cultivar “Tainong No. 20” (NN), suggesting that differential gene responsiveness may contribute to cultivar-specific cold tolerance. Additionally, most *AcLEA* genes exhibited distinct spatiotemporal expression patterns across floral organs and fruit at various developmental stages, suggesting their involvement in reproductive development. These findings provide a foundation for future functional studies and highlight candidate genes for improving cold resilience and developmental traits in pineapple through molecular breeding.

## Simple Summary

Late Embryogenesis Abundant (LEA) proteins are key for plant abiotic stress responses and development. Pineapple, a high-value tropical crop, faces cold stress threats, yet the research on *AcLEA* genes is scarce. This study identified 37 *LEA* genes in pineapple, classified into 6 subfamilies. Most AcLEA proteins are hydrophilic, thermally stable, and intrinsically disordered. Phylogenetic and collinearity analyses showed species-specific expansion mainly via segmental duplication, with some duplicated genes functionally diverging. Promoter *cis*-element, transcription factor, and microRNA network predictions indicated that *AcLEA* genes participate in stress responses and development. Expression profiling revealed that most of *AcLEA* genes were up-regulated under cold stress, implying they play roles in cold tolerance. Some *AcLEA* genes, such as *AcLEA32* and *AcLEA33*, were more strongly induced in cold-tolerant than sensitive cultivars. Most *AcLEA* genes had spatiotemporal expression in floral organs/fruits, suggesting these genes are involved in the reproduction process. This study may aid future studies and molecular breeding for cold-resilient pineapple.

## 1. Introduction

Plants encounter a range of abiotic and biotic challenges throughout their life cycles and have evolved various mechanisms to mitigate damage and ensure survival [1]. Among abiotic factors, drought, salinity, osmotic stress, cold, and freezing conditions may induce cellular dehydration, triggering the accumulation of highly hydrophilic Late Embryogenesis Abundant (LEA) proteins [2,3,4,5]. LEA proteins were first identified and characterized in cotton seeds [6] and are distinguished by high hydrophilicity, thermal stability, and intrinsic disorder, often enriched in glycine, lysine, and histidine residues [7]. According to sequence similarity and Pfam domain analysis, LEA proteins are classified into eight subgroups: LEA_1, LEA_2, LEA_3, LEA_4, LEA_5, LEA_6, dehydrin (DHN), and seed maturation protein (SMP) [8,9].

Functional studies have shown that LEA proteins act as “molecular shields,” preventing protein aggregation and preserving enzymatic activity during dehydration or freeze–thaw cycles [4,10]. Certain subfamilies (e.g., LEA_2, LEA_4) contain histidine-rich motifs that bind divalent cations and scavenge reactive oxygen species [11]. In planta, *LEA* genes are induced not only in desiccation-tolerant structures such as seeds and pollen but also in vegetative tissues (roots, leaves) and developing organs (flowers, fruits) under both normal and stress conditions [12,13]. Their expression is upregulated by drought, salinity, cold, heat, and oxidative stresses, implicating roles in osmotic protection, glass-state maintenance, ion homeostasis, and stress signaling [14,15,16,17,18]. Overexpression of the cucumber *CsLEA11* gene enhanced cell viability and conferred heat and cold tolerance in *Escherichia coli* [19]. Similarly, overexpression of maize *ZmLEA3* improved low-temperature stress tolerance in tobacco, yeast, and *E. coli* [20]. In tomato, *SiLEA4* significantly increased frost resistance by elevating antioxidant enzyme activity and proline content [21]. Expression of *MfLEA3* from alfalfa enhanced drought and cold tolerance in transgenic tobacco [22]. Overexpression of rice *OsLEA3-2* conferred tolerance to drought and salinity [23]. In tomato, *PsLEA4* overexpression regulated proline metabolism and antioxidant enzyme activity, resulting in improved cold adaptability and significantly higher yield compared with wild type [24]. Ectopic expression of *Arabidopsis LEA33* in *E. coli* enhanced cold stress tolerance, and in *Arabidopsis*, increased osmotic stress tolerance and abscisic acid sensitivity [25]. Moreover, expression of the *Arabidopsis* dehydrin *XERICO* in rice increased drought and salt tolerance by elevating ABA levels and ABA-mediated stress responses [26]. Collectively, these findings underscore the critical roles of LEA proteins in enhancing stress resilience and developmental regulation across diverse plant species.

Pineapple (*Ananas comosus* L.), a perennial herbaceous monocot in the Bromeliaceae family, is an economically and scientifically important tropical fruit [27]. Proper development of pineapple flowers and fruit is essential for yield and quality formation, yet chilling stress is a major constraint on year-round production [28,29,30,31,32,33]. While LEA proteins demonstrate cold tolerance enhancement across diverse plant species [19,21,22], tropical crops like pineapple face unique challenges. Unlike temperate species with natural cold acclimation mechanisms, tropical plants must rely primarily on constitutive protection systems. The specific roles of LEA proteins in tropical crop cold tolerance remain largely unexplored. Beyond stress responses, LEA proteins increasingly emerge as developmental regulators, particularly during reproductive phases characterized by natural dehydration stress (seed/pollen maturation) [34,35]. In tropical fruits like pineapple, where flower and fruit development spans extended periods under variable environmental conditions, LEA proteins may serve dual roles in both stress protection and developmental regulation.

The release of a high-quality pineapple genome provides an excellent opportunity to systematically characterize the *LEA* gene family. The relatively compact pineapple genome (~526 Mb) and its phylogenetic position among monocots make it an ideal model for understanding *LEA* evolution and function in tropical crops. Given the pronounced differences in cold tolerance among pineapple cultivars, we compared the cold stress responses of the tolerant cultivar “Comte de Paris” and the sensitive cultivar “Tainong 20” to identify *LEA* genes contributing to cultivar-specific stress responses and their potential applications in breeding programs. In this study, we performed a genome-wide identification and characterization of the *LEA* gene family in pineapple, and investigate its expression profiling during floral and fruit development and under cold stress based on transcriptome data and qRT-PCR analysis. These findings advance our understanding of *LEA* functions and provide a foundation for leveraging LEA-mediated mechanisms to improve cold tolerance in pineapple through molecular breeding.

## 2. Materials and Methods

### 2.1. Identification and Characterization of AcLEA Family in Pineapple

The genome and annotation files of pineapple (*Ananas comosus* L.) were downloaded from Phytozome (https://phytozome-next.jgi.doe.gov/info/Acomosus_v3, accessed on 20 May 2025) and protein database size (27,024 proteins). The Hidden Markov Model (HMM) profiles corresponding to LEA-related domains (PF03760, PF03168, PF03242, PF02987, PF00477, PF10714, PF04927, PF00257) were retrieved from the Pfam database (http://pfam.xfam.org/, accessed on 20 May 2025) and used to search the pineapple proteome using HMMER v3.3.2 with an E-value threshold of <1 × 10^−5^ and default parameters. Additionally, annotated LEA proteins from *Arabidopsis thaliana* (TAIR, https://www.arabidopsis.org/, accessed on 20 May 2025) and rice (*Oryza sativa*) (RGAP v7, https://rice.uga.edu, accessed on 20 May 2025) were used as queries in BLASTP v.2.14.0 searches against the pineapple protein database using the same E-value threshold. The results from HMMER and BLASTP were merged, and redundant sequences were manually removed. All putative LEA protein sequences were further validated for conserved domains using SMART (http://smart.embl-heidelberg.de/, accessed on 20 May 2025) and NCBI-CDD (https://www.ncbi.nlm.nih.gov/cdd, accessed on 20 May 2025) Only those sequences containing typical LEA-related domains (LEA, Dehydrin, or SMP) were retained, resulting in 37 non-redundant *AcLEA* genes.

### 2.2. Phylogenetic Analysis of AcLEAs

To investigate the evolutionary relationships of LEA proteins, a multiple sequence alignment of 37 pineapple AcLEA and 51 *Arabidopsis* AtLEA proteins [26] was performed using ClustalW v2.1 with default parameters [36]. A phylogenetic tree was constructed using FastTree with 1000 bootstrap replicates, applying the maximum likelihood method and the JTT amino acid substitution model [37]. The tree was visualized and annotated using iTOL v6.0 (https://itol.embl.de, accessed on 20 May 2025).

### 2.3. Gene Structure, Conserved Motif, and Cis-Regulatory Element Analysis of AcLEAs

The exon-intron structures and untranslated regions (UTRs) of *AcLEA* genes were extracted from the GFF3 genome annotation file. Conserved motifs were identified using the MEME Suite (https://meme-suite.org/tools/meme, accessed on 20 May 2025) with a maximum motif number of 10. For promoter analysis, the 2000 bp upstream genomic sequences from the start codon of each *AcLEA* gene were extracted using TBtools v2.149 [38]. *Cis*-regulatory elements were predicted using the PlantCARE database (http://bioinformatics.psb.ugent.be/webtools/plantcare/html/, accessed on 20 May 2025) and the visualization was conducted using TBtools and the R package “pheatmap” v1.0.13 (https://CRAN.R-project.org/package=pheatmap, accessed on 20 May 2025).

### 2.4. Three-Dimensional Structural Prediction of AcLEAs

The three-dimensional (3D) protein structures of AcLEA members were predicted by homology modeling using SWISS-MODEL (https://swissmodel.expasy.org/, accessed on 21 May 2025) [39]. Model quality was assessed based on GMQE (Global Model Quality Estimation, GMQE > 0.6, QMEAN4 Z-score between −2 and +2) scores. Structural visualization was performed using PyMOL v2.5.4 (https://pymol.org/2/, accessed on 21 May 2025). In addition, we conducted intrinsic disorder prediction using IUPred2A (https://iupred2a.elte.hu/, on 21 May 2025), and secondary structure prediction was performed using SOPMA (https://npsa.lyon.inserm.fr/cgi-bin/npsa_automat.pl?page=/NPSA/npsa_sopma.html, accessed on 21 May 2025) via the NPS@ server. To improve clarity, only one representative model per subfamily was displayed in the main figures, while all models were included in Figure S1.

### 2.5. Chromosomal Distribution and Gene Duplication Analysis of AcLEAs

The chromosomal locations of *AcLEA* genes were mapped using genome annotation data and visualized with Circos [40]. Segmental and tandem duplication events were detected using the MCScanX toolkit embedded in the JCVI module (accessed on 21 May 2025) [41]. MCScanX was run with default parameters, including a minimum of 5 genes per syntenic block and a maximum of 25 intervening genes allowed. Synteny relationships between pineapple and four representative species (two monocots: *Oryza sativa* and *Zea mays*; two dicots: *Arabidopsis thaliana* and *Vitis vinifera*) were analyzed to explore evolutionary conservation. The visualization of collinear blocks was performed using TBtools v2.3.6. To assess the selection pressure on duplicated gene pairs, Ka (nonsynonymous), Ks (synonymous) substitution rates, and Ka/Ks ratios were calculated using Ka/Ks_Calculator v3.0 [42] with default parameters. Gene pairs with Ka/Ks > 1 were considered to be under positive selection, while Ka/Ks < 1 indicated purifying selection.

### 2.6. Transcription Factor Binding Network Prediction

The putative promoter regions (−2000 bp upstream) of *AcLEA* genes were analyzed using the Plant Transcriptional Regulatory Map (PTRM) database (http://plantregmap.gao-lab.org/, accessed on 21 May 2025) to identify potential transcription factor (TF) binding sites, with *p*-value cutoff ≤ 1 × 10^−5^. The resulting TF–target interaction network was visualized using Cytoscape v3.10.0.

### 2.7. Plant Materials, Growth Conditions, and Cold Treatment

Pineapple growth is highly sensitive to temperature. Optimal development occurs at daily mean temperatures of 24–27 °C, while growth slows significantly at 10–14 °C and nearly ceases below 10 °C, while temperatures below 5 °C can cause chilling injury. As pineapple is cultivated near its northern distribution limits in regions such as Fujian and Guangxi, where winter temperatures may occasionally fall below 5 °C, the cold treatment conditions in this study were established based on field observations and represent realistic low-temperature stress scenarios. Cold-tolerant cultivar “Comte de Paris” (BL)and cold-sensitive cultivar “Tainong No. 20” (NN) were obtained from the Horticulture Research Institute, Guangxi Academy of Agricultural Sciences. BL and NN were cultivated in plastic pots under controlled conditions (24 °C, 60% humidity, 10 h light/14 h dark photoperiod, and 4000 lux light intensity). Plants were watered twice per week to maintain adequate soil moisture. After one week of acclimatization at 24 °C, cold treatment was initiated by transferring plants to a 5 °C environment. Three uniform, healthy pineapple plants per cultivar, each approximately 50 cm in height, were selected for cold stress treatment. After seven days at 5 °C, they were returned to 24 °C to relieve the stress. Samples were collected at 0 h, 4 h, 24 h, and 7 days during the cold treatment, and at 4 h, 24 h, and 7 days after recovery at 24 °C, for transcriptome and quantitative real-time PCR (qRT-PCR) analyses. RNA-seq data have been deposited in the China National GeneBank DataBase (CNGBdb) under accession number CNP0008093.

### 2.8. RNA Extraction and qRT-PCR Analysis

Total RNA was extracted from cold-treated samples using the TRIzol reagent (Invitrogen, Waltham, MA, USA). And RNA integrity and purity were evaluated using 1% agarose gel electrophoresis and Nanodrop spectrophotometry, ensuring 260/280 ≥ 1.8 and 260/230 ≥ 2.0. First-strand cDNA synthesis was carried out using the ThermoScript RT-PCR system (Thermo Fisher Scientific, Waltham, MA, USA). Primers for *AcLEA* genes (length of 18–25 bp and GC content of 50–60%) were designed using IDT SciTools Web Tools (https://sg.idtdna.com/pages/tools/, accessed on 21 May 2025) and are listed in Table S9. qRT-PCR was performed with SYBR Premix Ex Taq II (TaKaRa, Dalian, China) on a Bio-Rad CFX96 Real-Time PCR System (Bio-Rad, Hercules, CA, USA). Thermal cycling conditions: 95 °C for 30 s, 40 cycles of 95 °C for 5 s, and 60 °C for 30 s, followed by 95 °C for 15 s for melting curve analysis. Three independent biological replicates were included for each treatment, and each biological sample was subjected to three technical repeats. Statistical significance of qRT-PCR data was assessed using two-way ANOVA followed by Tukey’s post hoc test (*p* ≤ 0.05). Expression levels were log_2_-transformed before analysis. For genome-wide comparisons, multiple testing correction was performed using the Benjamini–Hochberg method (FDR ≤ 0.05). Relative expression levels were calculated using the CT (2^−ΔΔCT^) method [43,44].

## 3. Results

### 3.1. Genome-Wide Identification and Basic Features of the AcLEA Gene Family

To systematically identify LEA family members in pineapple (*Ananas comosus*), we performed a genome-wide search using a combination of HMM search and BLASTP alignment. A total of 37 *AcLEA* genes were identified and named *AcLEA1* to *AcLEA37* based on their chromosomal positions (Table S1). These *AcLEA* genes encode proteins ranging in length from 89 amino acids (AcLEA1 and AcLEA27) to 494 amino acids (AcLEA29), with corresponding molecular weights between 9.31 kDa and 54.01 kDa. The predicted isoelectric points (pI) of the AcLEA proteins ranged from 4.91 (AcLEA30) to 11.38 (AcLEA4), indicating that the AcLEA family includes both acidic and basic proteins, which may reflect their functional diversity in different cellular environments. The instability index analysis revealed that the majority of AcLEA proteins may be unstable in vitro, particularly AcLEA1, AcLEA10, and AcLEA25, which had the highest values. The aliphatic index ranged from 29.66 (AcLEA27) to 120.19 (AcLEA21), and seven proteins (AcLEA5, AcLEA12, AcLEA17, AcLEA20, AcLEA21, AcLEA32, and AcLEA35) showed values greater than 100, suggesting strong potential for heat stability and possible roles under thermal or dehydration stress. The GRAVY (grand average of hydropathicity) scores revealed that approximately two-thirds of AcLEA proteins (24 out of 37) had negative values, indicating a predominantly hydrophilic nature. This aligns with the known characteristics of LEA proteins, which are typically water-soluble and function in stress-protective mechanisms through hydration buffering and membrane stabilization. Subcellular localization predictions suggested that AcLEAs are broadly distributed in various compartments, including the nucleus (nucl), cytoplasm (cyto), chloroplast (chlo), mitochondrion (mito), plasma membrane (plas), vacuole (vacu), endoplasmic reticulum (E.R.), and peroxisome (pero), which may reflect their functional diversity in cellular protection under stress conditions.

### 3.2. Phylogenetic Analyses of the AcLEA Genes

**Figure 1:**
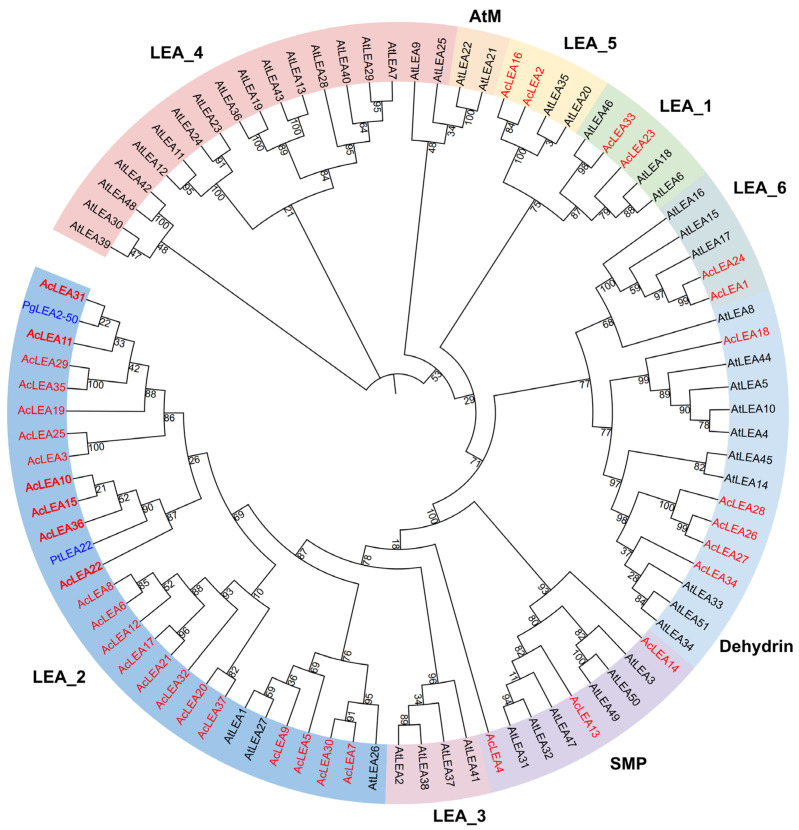
Unrooted maximum-likelihood (ML) phylogenetic tree of LEA proteins from *Arabidopsis thaliana* (At), *Ananas comosus* (Ac), and 2 reported LEA proteins involved in stress resistance. AtLEAs, AcLEAs, and the reported PgLEA2-50 and PtLEA22 are highlighted with black, red, and blue color, respectively.

To investigate the evolutionary relationships of AcLEAs, a phylogenetic tree was constructed using the LEA protein sequences from pineapple (37), *Arabidopsis thaliana* (51), and two reported stress-related LEA proteins (PgLEA2-50 and PtLEA22) (Figure 1 and Table S2) [45,46]. Most LEA subfamily clades in the phylogenetic tree are supported by moderate to high bootstrap values (generally >70%), indicating that the overall classification is reasonably robust. All the LEA proteins were classified into nine subfamilies: LEA_1, LEA_2, LEA_3, LEA_4, LEA_5, LEA_6, Dehydrin, SMP, and AtM. Compared to *Arabidopsis*, in which all nine subfamilies were represented, the AcLEAs were distributed across six subfamilies, with no representatives in LEA_3, LEA_4, and AtM. Notably, the LEA_2 subfamily was the most expanded in pineapple, containing 23 genes (62.16% of all AcLEAs). PgLEA2-50 and PtLEA22, previously shown to enhance cold tolerance [45,46], clustered closely with AcLEA11, AcLEA31, AcLEA10, AcLEA15, AcLEA22, and AcLEA36, indicating that these pineapple genes may also participate in cold stress adaptation.

### 3.3. Gene Structure, Conserved Motif, and Domain Analysis of AcLEAs

**Figure 2:**
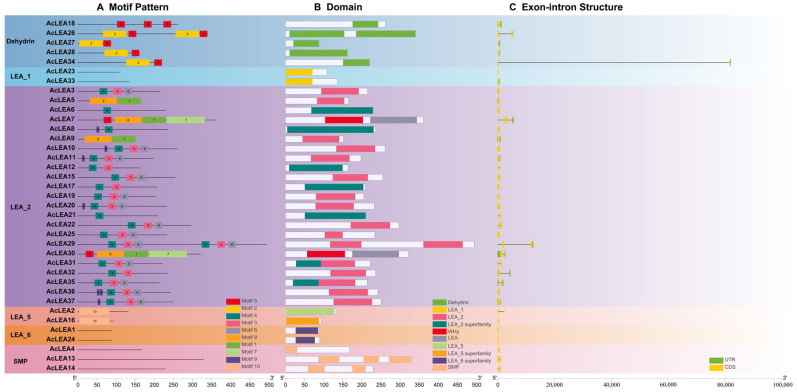
Motif compositions, conserved domains and gene structures of *AcLEA* genes. (**A**) Distribution of conserved motifs in AcLEA proteins. Different boxes represent different motifs. (**B**) Distribution of conserved domains in AcLEA proteins. Different domains are displayed by boxes with different colors. (**C**) The structure of *LEA* genes in Pineapple. The green boxes represent untranslated regions (UTRs), the yellow boxes represent exons, and the black lines represent introns.

To examine the structural diversity of *AcLEAs*, the gene exon-intron structures, conserved protein motifs, and functional domains were analyzed. The conserved motifs of 37 AcLEA proteins were predicted using the MEME tool (Figure 2A). Within the LEA_2 subfamily, all members, except *AcLEA5*, *AcLEA7*, *AcLEA9*, and *AcLEA30*, contained motif 4. Proteins in the LEA_5 subfamily exclusively contained motif 10. In the Dehydrin subfamily, all members harbored both motif 2 and motif 5, except *AcLEA18*, which only contained motif 5. Additionally, motif prediction was not applicable to the LEA_1 (*AcLEA23*, *AcLEA33*), LEA_6 (*AcLEA1*, *AcLEA24*), and SMP (*AcLEA4*, *AcLEA13*, *AcLEA14*) subfamilies. Conserved domain analysis (Figure 2B) showed that members of the LEA_1, LEA_6, SMP, and Dehydrin subfamilies possessed LEA_1, LEA_6 superfamily, SMP, and Dehydrin domains, respectively. Most proteins in the LEA_2 subfamily carried LEA_2 or LEA_2 superfamily domains, while *AcLEA7* and *AcLEA30* contained WHy and LEA domains. LEA_5 subfamily members had LEA_5 or LEA_5 superfamily domains. Overall, proteins within the same subfamily exhibited similar motif compositions and conserved domain characteristics and the absence of detectable motifs in certain subfamilies likely reflects high sequence divergence.

The analysis of exon–intron structures revealed that the majority of *AcLEA* genes exhibited incomplete gene structures (Figure 2C). Specifically, 21 *AcLEA* genes lacked both 5′ and 3′ untranslated regions (UTRs), 2 embodied only a 5′UTR, 6 contained only a 3′UTR, and the remaining 8 possessed complete UTRs. The number of exons ranged from 1 to 4. Consistent with findings in other plant species [45,47], several *AcLEA* genes were found to be intron-less, including members of the LEA_1 (*AcLEA23*), LEA_2 (*AcLEA3*, *AcLEA6*, *AcLEA8*, *AcLEA10*, *AcLEA17*, *AcLEA19*, *AcLEA25* and *AcLEA36*) and LEA_6 (*AcLEA1* and *AcLEA24*) subfamilies were intron-less, which may reflect evolutionary selection for rapid gene expression under stress.

### 3.4. Protein Model Prediction of AcLEAs

**Figure 3:**
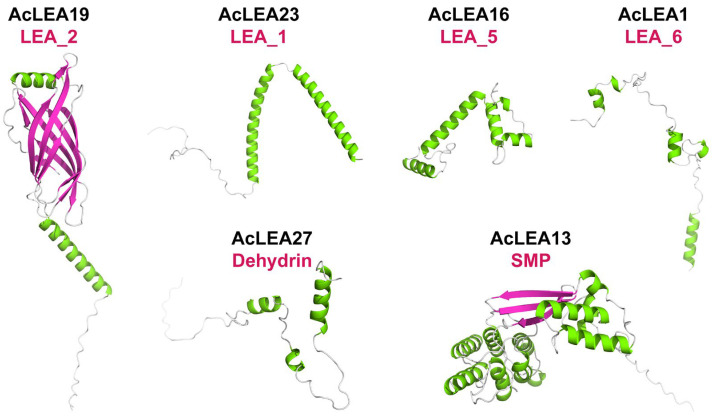
Predicted 3D structures modeling of representative AcLEA proteins. The protein structure with the highest GMQE scores in each subfamily was selected as the representative model.

To gain insight into the structural properties of pineapple LEA proteins, both secondary structure prediction and 3D modeling were performed for all 37 AcLEA proteins. The secondary structure analysis revealed that random coil was the dominant component across most AcLEA proteins, with 24 members exhibiting more than 50% random coil content (Figure S1 and Table S3). This high proportion of intrinsically disordered regions is consistent with the well-established role of LEA proteins as flexible “molecular shields” during cellular stress. This suggests that a majority of AcLEA proteins are intrinsically disordered, a common feature of stress-responsive LEA proteins [48]. Notably, AcLEA26, AcLEA28, and AcLEA1 showed the highest random coil proportions (>75%), whereas members of the SMP and LEA_1 subfamilies, such as AcLEA14 and AcLEA33, exhibited higher α-helix content, indicating more ordered structural features. Such relatively more ordered regions may imply subfamily-specific structural specialization and the potential for specific protein–protein interactions, although these possibilities require future experimental validation. The LEA_2 subfamily displayed the greatest diversity in secondary structure composition, including variable contents of α-helices, β-strands, and disordered regions, highlighting potential functional divergence among its members. To visualize the structural diversity among LEA subfamilies, the highest-scoring member from each subfamily was selected for 3D structure modeling (Figure 3). The resulting structures reflected clear differences across subfamilies. For example, AcLEA19 (LEA_2) exhibited a β-barrel-like fold composed of extended strands and helices, while AcLEA23 (LEA_1), AcLEA16 (LEA_5), and AcLEA27 (Dehydrin) were dominated by α-helical elements. AcLEA1 (LEA_6) showed a loosely coiled and highly disordered structure, whereas AcLEA13 (SMP) presented a well-organized architecture comprising both helices and strands. Given the predominantly disordered nature of LEA proteins, the 3D models should be interpreted cautiously, as current algorithms have limited reliability for IDPs and likely capture only the more stable or stress-induced structural elements. Together, these results indicated that AcLEA proteins possess diverse structural conformations across different subfamilies, which may be linked to their distinct functional roles under stress conditions.

### 3.5. Cis-Regulatory Elements Identification in AcLEA Promoters

**Figure 4:**
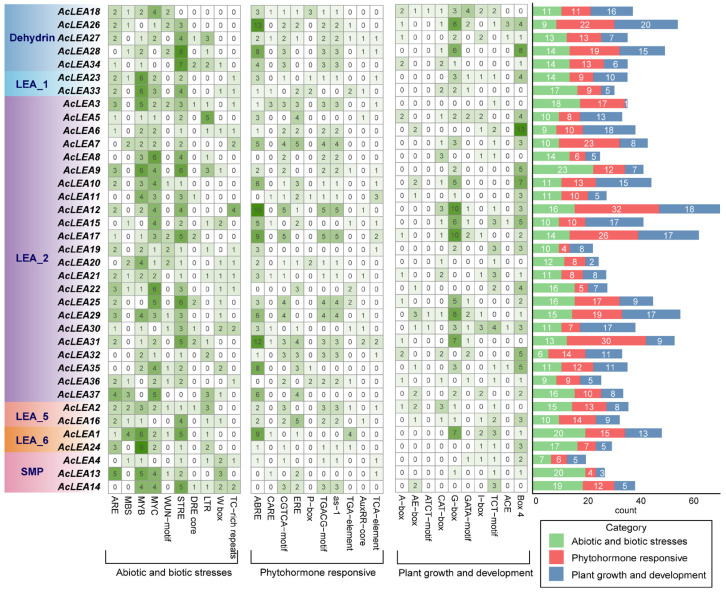
The number and type of predicted *cis*-elements in the putative promoter region of *AcLEA* genes.

To explore the potential regulatory mechanisms underlying the expression of *AcLEA* genes, we analyzed *cis*-regulatory elements within the 2000 bp upstream promoter regions of all 37 members. The identified elements were classified into three major functional categories: abiotic and biotic stress-responsive, phytohormone-responsive, and plant growth and development-related elements (Figure 4 and Table S4). The most abundant *cis*-elements in these three categories were STRE (108), ABRE (143) and G-box (104), respectively. Among stress-responsive elements, STRE and LTR (low-temperature responsive) motifs were found in the promoters of 35 and 20 genes, respectively, indicating that many *AcLEA* genes may be involved in cold stress responses. Additionally, TC-rich repeats, which are associated with defense and drought responses, were frequently observed in the LEA_2 and SMP subfamilies. Regarding hormone-related elements, ABRE (ABA-responsive element) was detected in 31 genes, and CGTCA-motif (methyl jasmonate-responsive) was found in 27 genes. ABRE was particularly enriched in the Dehydrin and LEA_5 subfamilies, suggesting possible involvement in ABA-mediated stress signaling. For plant growth and development related elements, light-responsive elements, such as G-box (26 genes), TCT-motif (23 genes) and Box 4 (29 genes) were present in most AcLEA genes. Notably, G-box elements were particularly abundant in the Dehydrin and LEA_1 subfamilies, indicating potential light-regulated expression. Moreover, certain elements occurred in specific subfamilies. For instance, ACE and ATCT-motif elements were exclusively found in the LEA_2 and Dehydrin subfamilies. Overall, the diversity and abundance of *cis*-regulatory elements suggest that *AcLEA* genes were regulated by a complex transcriptional network involving environmental stimuli, hormone signals, and developmental cues. Subfamily-specific *cis*-regulatory element enrichment patterns further imply possible functional specialization among LEA subgroups in pineapple.

### 3.6. Chromosomal Distribution, Collinearity and Evolution Analysis of AcLEAs

**Figure 5:**
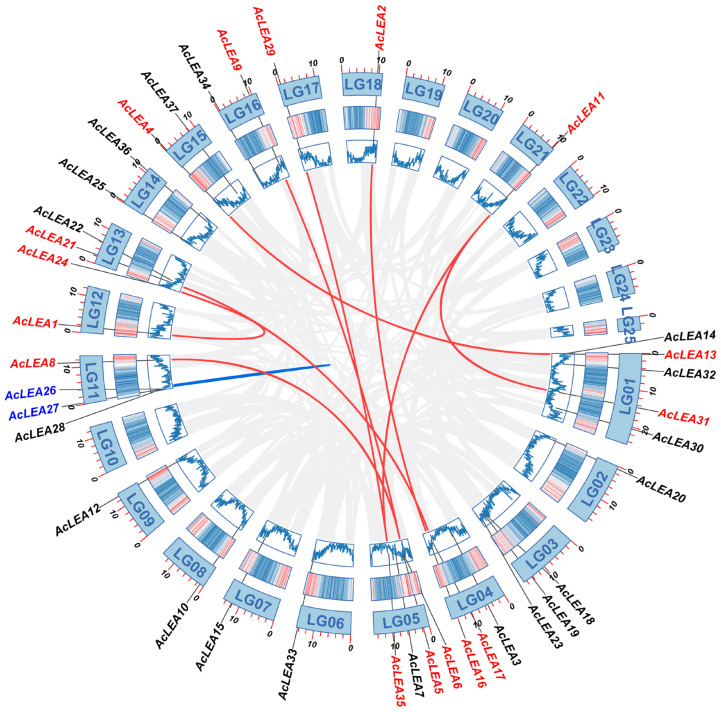
Distribution and collinearity of *AcLEA* genes in the pineapple genome. The background gray lines represent all the syntenic blocks in the pineapple genome. The red lines represent duplicated *AcLEA* gene pairs, and the blue line represents tandem repeat gene pair.

All 37 *AcLEA* genes were unevenly distributed across 18 chromosomes. Specifically, five genes were located on LG01, four genes each on LG05 and LG11, and three genes on LG03, LG04, and LG13, respectively. Two genes were found on LG14, LG15, and LG16, while nine linkage groups (LG02, LG06, LG07, LG08, LG09, LG12, LG17, LG18, and LG21) each harbored only one *AcLEA* gene. The expansion of gene families is often driven by duplication events, which are considered key forces in species evolution [49]. To investigate the potential mechanisms underlying the expansion of the *AcLEA* gene family, a collinearity analysis was conducted across the pineapple genome. A total of nine segmental duplication events and one tandem duplication event were identified, suggesting that segmental duplication played a major role in the evolution of the *AcLEA* gene family (Figure 5 and Table S5). Six of the segmentally duplicated gene pairs belonged to the LEA_2 subfamily, including *AcLEA31/AcLEA11*, *AcLEA17/AcLEA21*, *AcLEA6/AcLEA8*, *AcLEA5/AcLEA9*, *AcLEA35/AcLEA29*, and *AcLEA35/AcLEA11*. Additionally, *AcLEA16/AcLEA2*, *AcLEA1/AcLEA24*, and *AcLEA13/AcLEA4* were identified as duplicated gene pairs from the LEA_5, LEA_6, and SMP subfamilies, respectively. The single tandem duplication event (*AcLEA26/AcLEA27*) occurred within the Dehydrin subfamily. To assess the evolutionary pressures acting on these duplicated genes, the non-synonymous to synonymous substitution rate ratios (Ka/Ks) were calculated for ten duplicated gene pairs (Table S5). Ka/Ks values were obtained for all pairs, except *AcLEA26/AcLEA27*. Among them, the Ka/Ks ratio for *AcLEA35/AcLEA11* was 1.38, suggesting that this gene pair underwent strong positive selection. The Ka/Ks ratios for all other gene pairs were less than 1, indicating that the *AcLEA* gene family has predominantly evolved under purifying selection.

**Figure 6:**
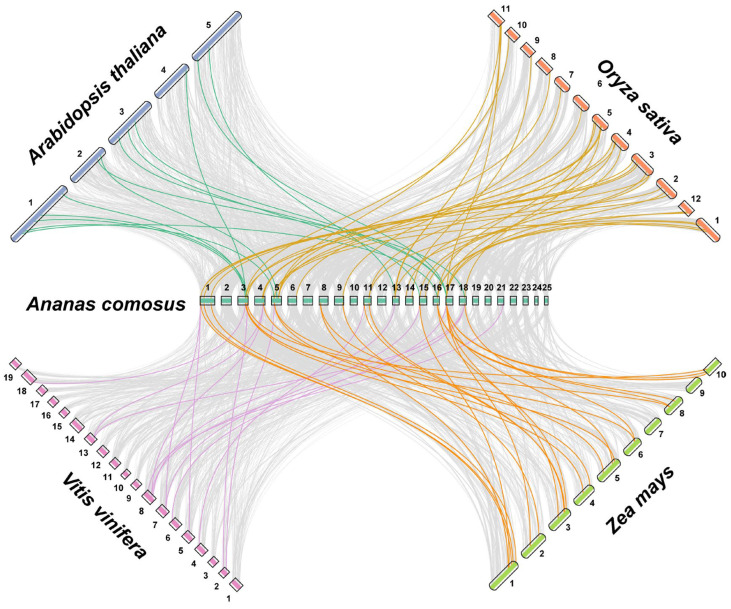
Collinear analysis of *LEA* genes from pineapple and four representative species. Gray lines indicate collinear blocks in pineapple and other genomes, while colorful lines highlight syntenic *LEA* gene pairs. *Arabidopsis thaliana*, *Vitis vinifera*, *Oryza sativa*, and *Zea mays* refer to *Arabidopsis*, grape, rice, and maize, respectively.

Collinearity analysis, which compares the conserved gene order across different species, provides important insights into gene family evolution and the retention of ancestral genomic blocks [50]. In this study, syntenic relationships were examined between pineapple and four representative plant species: two dicots (*Arabidopsis thaliana* and *Vitis vinifera*) and two monocots (*Oryza sativa* and *Zea mays*) (Figure 6 and Table S6). The analysis revealed varying degrees of collinearity between the *AcLEA* genes and those of the other species. A total of 16 collinear gene pairs were identified between pineapple and *Arabidopsis*, 16 with grape, 33 with rice, and 28 with maize, indicating a relatively closer evolutionary relationship between pineapple and monocot species. Notably, five *AcLEA* genes—*AcLEA18* (Dehydrin), *AcLEA29* (LEA_2), *AcLEA2* (LEA_5), *AcLEA23* (LEA_1), and *AcLEA4* (SMP)—were found to share collinear relationships with all four species, suggesting that these genes may represent evolutionarily conserved core members of the *LEA* gene family that originated prior to the divergence of monocots and dicots. In contrast, no collinear orthologs were identified for the LEA_6 subfamily in any of the four species, which may indicate lineage-specific expansion or rapid diversification of LEA_6 genes in pineapple.

### 3.7. Transcription Factor Regulatory Network of AcLEA Genes

**Figure 7:**
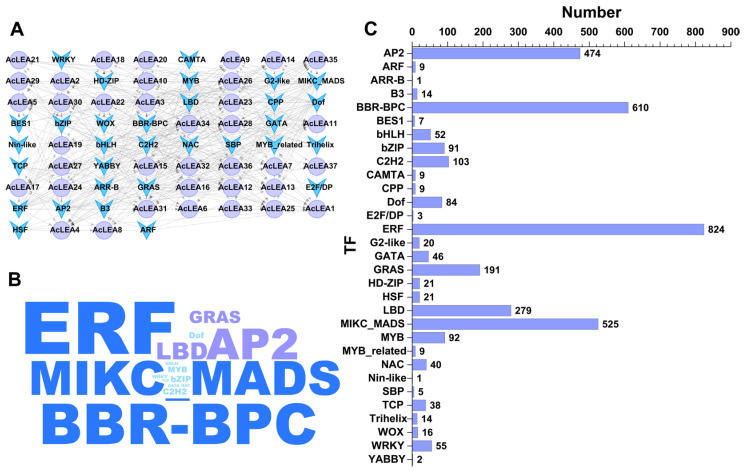
The predicted transcription factor regulatory network of *AcLEA* genes in pineapple. (**A**) The transcriptional regulatory network of *AcLEA* genes, where purple circular nodes represent *AcLEAs*, and blue arrow-shaped ones denote transcription factors; (**B**) The word cloud map of the transcription factors; (**C**) Quantity statistics of the transcription factors.

To gain a comprehensive understanding of the regulatory mechanisms controlling *AcLEA* gene expression, transcription factors (TFs) potentially targeting the promoter regions of the 37 *AcLEA* genes were predicted using the Plant Transcriptional Regulatory Map (PlantRegMap) database. The results revealed that *AcLEA* genes were regulated by a diverse array of TF families, including ERF, BBR-BPC, MIKC_MADS, AP2, and LBD (Figure 7 and Table S7). Among them, ERF was the most abundant, with a total of 824 predicted binding events, followed by BBR-BPC (610) and MIKC_MADS (525). Most *AcLEA* genes were predicted to be targets of ERF TFs, especially *AcLEA34*, which had the highest number of predicted TF binding sites (548), including 383 ERF sites. Other ERF-regulated genes included *AcLEA31*, *AcLEA32*, and *AcLEA28*, indicating a strong regulatory influence of the ERF family on the *AcLEA* gene network. In addition to ERF, several other TF families associated with abiotic stress responses were identified, such as AP2 and CAMTA (cold stress), and NAC, Dof, and MYB (drought stress) [51]. Moreover, TF families related to plant growth and development, such as MIKC_MADS, GRAS, and bZIP, were also detected, implying that *AcLEA* genes may also be involved in developmental regulatory pathways in addition to their stress-related functions.

### 3.8. miRNA-Mediated Regulatory Mechanisms of AcLEA Genes

**Figure 8:**
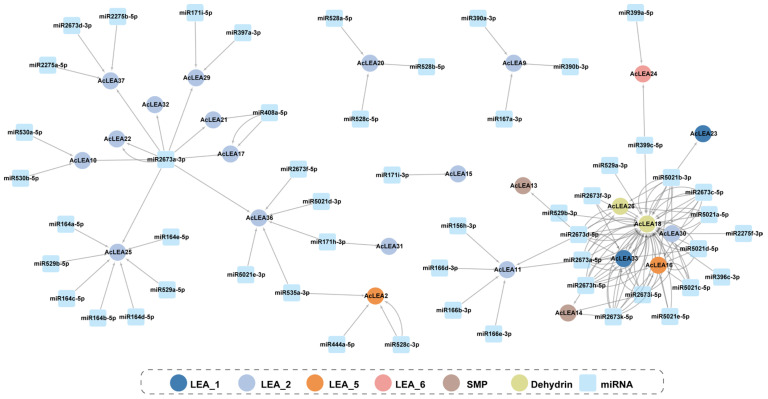
miRNA network diagram targeting pineapple *LEAs*. The light blue square nodes represent the predicted miRNAs, and the colored round nodes represent the targeted *AcLEA* genes.

MicroRNAs (miRNAs) are 19–25 nt non-coding RNAs that regulate gene expression post-transcriptionally by directing RNA-induced silencing complexes (RISC) to target mRNAs [52]. To explore the potential post-transcriptional regulation of *AcLEA* genes in pineapple, we predicted miRNA-target interactions based on known plant miRNA datasets. A total of 143 miRNAs were predicted to target 25 *AcLEA* genes, forming a complex regulatory network (Figure 8 and Table S8). Among the *AcLEA* genes, *AcLEA18* (LEA_1) had the most miRNA target sites (53), followed by *AcLEA33* (LEA_1) with 13, indicating that *AcLEA18* might be under tight post-transcriptional control and involved in diverse regulatory pathways. In terms of miRNA families, miR2673 was the most prevalent, with 72 predicted binding events, followed by the miR5021 family (30 sites). Notably, miR2673a-3p was predicted to target nine genes from the LEA_2 subfamily, indicating a subfamily-specific regulatory preference. Genes such as *AcLEA26*, *AcLEA18*, *AcLEA30*, *AcLEA33*, and *AcLEA16* were co-targeted by members of both miR2673 and miR5021 families, forming a dense regulatory network. These findings suggest that the *AcLEA* gene family was subject to complex miRNA-mediated post-transcriptional regulation, which might play a crucial role in coordinating their functions during stress adaptation and developmental processes in pineapple.

### 3.9. Expression Profile of AcLEAs in Different Tissues

**Figure 9:**
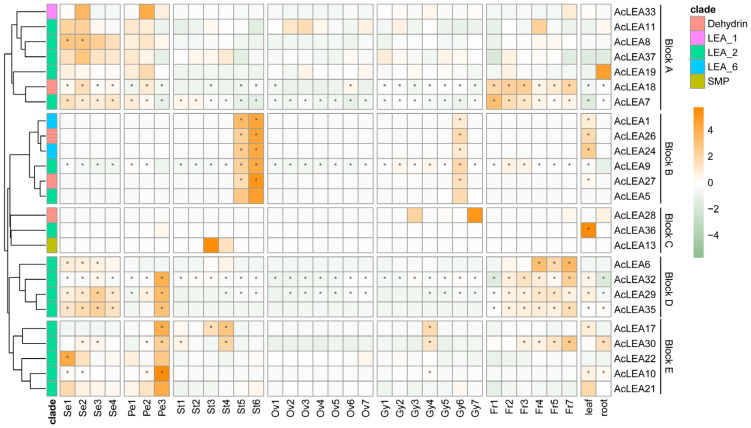
Tissue-specific expression profiles of *LEA* genes in pineapple. The heatmap is created based on the log_2_ (TPM + 0.01) expression values of *AcLEAs* and normalized by row. The TPM value higher than 50 is shown as abundant genes and marked with “*”. The orange indicates high gene expression and the green indicates low gene expression. Se: sepal; Pe, petal; St, stamen; Gy: Ov, ovule; gynoecium; Fr: fruit; the numbers represent different developmental periods, and the higher the number, the later the period.

To understand the possible role of *AcLEA* genes in pineapple growth and development, we analyzed the expression patterns of *AcLEAs* in different organs (root, leaf, flower and fruit) and floral tissues at different stages of development (sepal: stages Se1 to Se4, gynoecium: stages Gy1 to Gy7, ovule: stages Ov1 to Ov7, petal: stages Pe1 to Pe3 and stamen: stages St1 to St6), using RNA-seq data. After filtering low-expression genes, hierarchical clustering grouped the remaining *AcLEA* members into five distinct expression blocks (A-E) (Figure 9). Specifically, genes clustered in block A (e.g., *AcLEA33*, *AcLEA11*, *AcLEA8*, *AcLEA37*, *AcLEA19*, *AcLEA18*, *AcLEA7*) were predominantly expressed during sepal development and the early stages of petal formation (Pe1-Pe2). Genes clustered in block B, including *AcLEA1*, *AcLEA26*, *AcLEA24*, *AcLEA9*, *AcLEA27*, *AcLEA5*, were mainly active in late stamen development (St5-St6), at a specific late gynoecium stage (Gy6), and in leaves. Block C contained only a few genes with highly specific expression, such as *AcLEA28*, which peaked at Gy3 and Gy7 of gynoecium development, *AcLEA36*, which was strongly expressed in leaf, and *AcLEA13*, which showed elevated expression in mid-stamen stages (St3-St4). In contrast, block D genes (e.g., *AcLEA6*, *AcLEA32*, *AcLEA29*, *AcLEA35*) were broadly expressed across multiple tissues, exhibiting high levels during sepal formation, late petal development (Pe3), and a steadily increasing trend throughout fruit maturation (Fr1-Fr7); except for *AcLEA6*, these genes also displayed abundant expression in leaf and root. Block E genes (e.g., *AcLEA17*, *AcLEA30*, *AcLEA22*, *AcLEA10*, *AcLEA21*) were primarily enriched in early sepal and late petal developmental stages. Moreover, certain genes such as *AcLEA7*, *AcLEA9*, and *AcLEA32* exhibited high expression across nearly all surveyed tissues and developmental stages, suggesting that they may participate widely in various biological processes in pineapple.

### 3.10. Expression Patterns of AcLEA Genes in Response to Clod Stress

**Figure 10:**
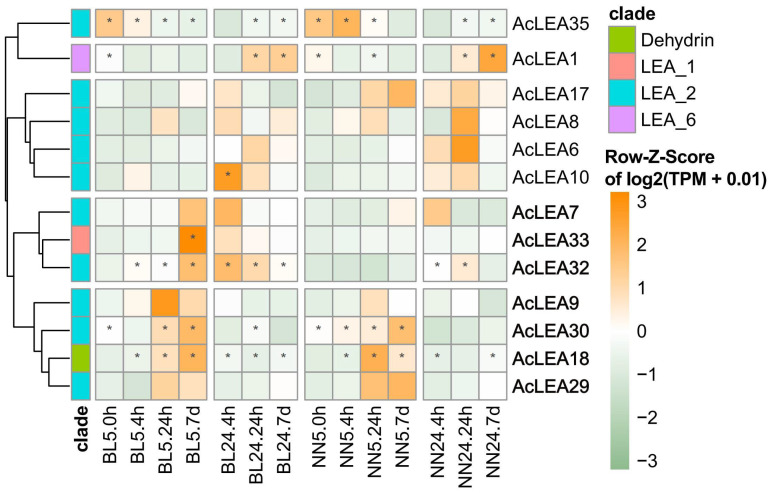
Expression profiles of *AcLEAs* in BL and NN during cold treatment at 5 °C (0 h, 4 h, 24 h, and 7 d). The heatmap was created from the log_2_(TPM + 0.01) values of *AcLEAs* and normalized by row. The TPM values above 50 are shown as high-abundance genes and are marked with “*”. Differences in gene expression changes are shown in color as the scale, with orange for high expression and green for low expression. BL is the cold-tolerant cultivar “Comte de Paris” and NN is the sensitive cultivar “Tainong No. 20”.

Pineapple is a major economic fruit crop in tropical and subtropical and routinely endured periodic winter cold events that led to yield reduction and economic loss [53]. We investigated the expression patterns of *AcLEA* genes during exposure to 5 °C low-temperature stress and following recovery at 24 °C using transcriptome data. After filtering out genes with consistently low expression, hierarchical clustering revealed that most *AcLEA* genes displayed dynamic expression patterns under cold stress conditions and recovery treatments (Figure 10). Among these, *AcLEA35* and *AcLEA1* exhibited decreased expression under cold stress in both cultivars, followed by gradual expression recovery upon return to normal temperatures. In contrast, most of the other *AcLEA* genes, including *AcLEA9*, *AcLEA30*, *AcLEA18*, and *AcLEA29* showed a progressive increase in expression levels with extended cold exposure, peaking at later stages of the cold treatment and rapidly decreasing after recovery. Genes such as *AcLEA7, AcLEA33*, and *AcLEA32* were also highly expressed towards the late stage of cold treatment but maintained elevated levels into the early recovery period before declining. Additionally, *AcLEA10* and *AcLEA6* were primarily induced during the early recovery phase. Although the general expression trends of *AcLEA* genes under cold stress and recovery were similar between BL and NN, their response timing and magnitude differed. Notably, genes such as *AcLEA7*, *AcLEA33*, and *AcLEA32* showed earlier and stronger induction in the cold-tolerant cultivar BL compared to the cold-sensitive cultivar NN, especially in the late cold-stress and early recovery phases. These findings suggested that multiple *AcLEA* genes might be involved in cold stress response in pineapple, with differential expression patterns potentially contributing to the distinct cold tolerance observed between cultivars.

**Figure 11:**
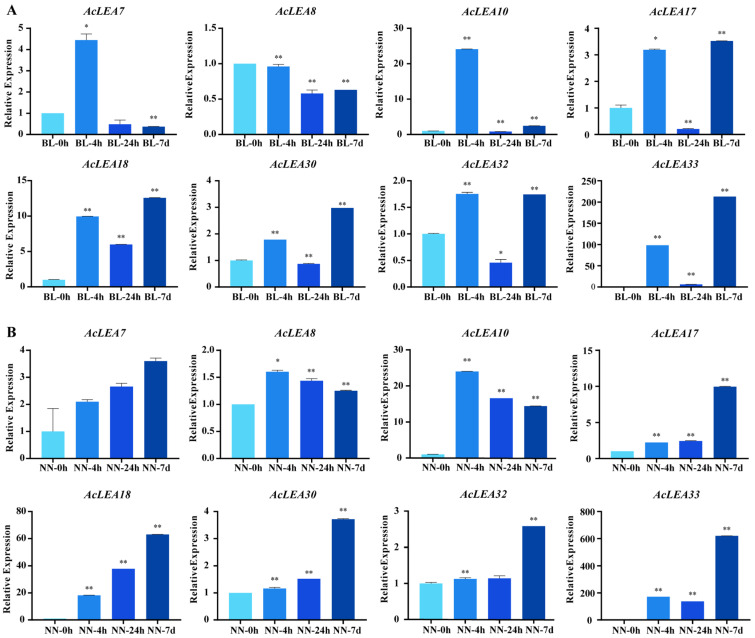
qRT-PCR analysis of eight genes (*AcLEA7*, *AcLEA8*, *AcLEA10*, *AcLEA17*, *AcLEA18*, *AcLEA30*, *AcLEA32*, and *AcLEA33*) under 5 °C cold stress treatment. (**A**) qRT-PCR analysis of *AcLEA7*, *AcLEA8*, *AcLEA10*, *AcLEA17*, *AcLEA18*, *AcLEA30*, *AcLEA32* and *AcLEA33* in BL cultivars under cold stress, BL for the cold-tolerant cultivar “Comte de Paris”; (**B**) qRT-PCR analysis of *AcLEA7*, *AcLEA8*, *AcLEA10*, *AcLEA17*, *AcLEA18*, *AcLEA30*, *AcLEA32* and *AcLEA33* in NN cultivars under cold stress, NN for the sensitive cultivar “Tainong No. 20”. Significant differences were analyzed by Student’s *t*-test (* *p*-value < 0.05 and ** *p*-value < 0.01).

To further validate the cold-responsive expression patterns of *AcLEA* genes, we selected eight representative *AcLEA* members and performed qRT-PCR analysis under the same batch of 5 °C cold treatment conditions (Figure 11). These genes included six from the LEA_2 subfamily, one from the Dehydrin subfamily (*AcLEA18*), and one from the LEA_1 subfamily (*AcLEA33*). Consistent with the transcriptome results, most *AcLEA* genes were upregulated following cold treatment; however, their expression dynamics differed between the two cultivars. In the cold-tolerant cultivar BL, all *AcLEA* genes except *AcLEA8* responded rapidly to low temperature, showing significant induction at 4 h post-treatment. In contrast, in the cold-sensitive cultivar NN, only *AcLEA8* and *AcLEA10* exhibited early induction, peaking at 4 h. The remaining genes showed delayed responses, with higher expression levels observed at 7 days post treatment. Specifically, *AcLEA7* and *AcLEA18* displayed a gradually increasing trend over time, while *AcLEA17*, *AcLEA30*, *AcLEA32*, and *AcLEA33* reached their highest expression levels during the late stages of cold treatment.

## 4. Discussion

The *LEA* (Late Embryogenesis Abundant) gene family is widely distributed across plant species and is known to play critical roles in protecting cells from abiotic stress and in regulating various growth and developmental processes [54,55,56]. In the present study, 37 *LEA* genes were identified in pineapple (*Ananas comosus*) and were named *AcLEA1* to *AcLEA37* based on their chromosomal locations (Table S1). Although the raw gene number is comparable to tomato (27) [57], potato (29) [58], and Chinese plum (30) [59], considering the relatively compact pineapple genome (~526 Mb), the 37 *AcLEA* genes represent a higher gene density (0.07 genes/Mb) compared to species like tomato (0.03 genes/Mb, the genome size is about 859.9 Mb) [60], indicating potential species-specific adaptation to environmental stress. Such differences in *LEA* gene numbers among species may result from variations in genome size, ploidy level, gene duplication frequency, and species-specific evolutionary pressures [61]. 

**Figure 12:**
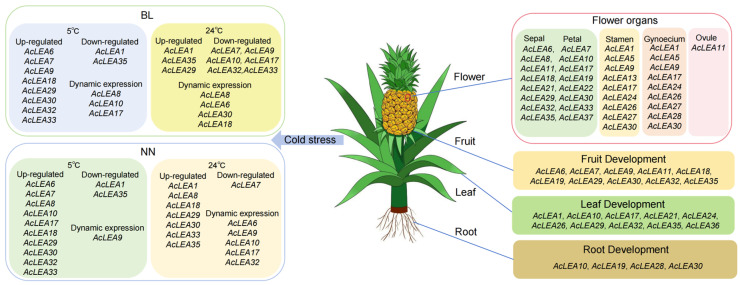
Schematic model of pineapple and expression patterns of *AcLEA* genes and its response to cold stress. The genes listed were highly expressed genes under the corresponding tissue and treatment.

Phylogenetic analysis of pineapple and *Arabidopsis LEA* proteins classified these genes into nine subfamilies (Figure 1). However, only six of these subfamilies were represented in pineapple, with no members identified in the LEA_3, LEA_4, or AtM subfamilies. In most plant species, LEA proteins are typically categorized into 6–8 subfamilies, with LEA_1, LEA_2, Dehydrin, and SMP considered as core subgroups. However, species-specific gene loss or expansion events have led to the absence or overrepresentation of certain subfamilies in different plants [62,63,64,65,66]. For example, in *Panax notoginseng*, the LEA_6 subfamily was absent [47]. These observations suggest that the evolution of the *LEA* gene family exhibits species-specific characteristics. In pineapple, most members belonged to the LEA_2 subfamily (accounting for 62.3%), a trend also observed in tomato [67], *Populus trichocarpa* [63], and *Capsicum annuum* L. [64]. The absence of LEA_3, LEA_4, and AtM in pineapple contrasts with their presence in rice and *Arabidopsis*, possibly reflecting evolutionary loss or functional compensation by the expanded LEA_2 subfamily. However, this contrasts with rice [68] and *Brassica napus* [66], where the largest subfamilies were Dehydrin and LEA_4, respectively, indicating substantial variation in subfamily composition across species. Collinearity analysis revealed that the number of collinear *LEA* gene family members between pineapple and monocot genomes (including rice and maize) was greater than that between pineapple and dicot genomes (including grape and *Arabidopsis*), supporting rice and maize as appropriate models for monocot stress gene function studies (Figure 6 and Table S6). The higher synteny with monocots (rice: 33, maize: 28) compared to dicots (*Arabidopsis*: 16, grape: 16) supports the use of rice and maize as superior models for functional validation of pineapple LEA genes, particularly in understanding monocot-specific stress adaptations. Some *AcLEA* genes including *AcLEA18* (Dehydrin) and *AcLEA29* (LEA_2) were collinear with genes in all four species. Expression analysis indicated that *AcLEA18* and *AcLEA29* were broadly expressed in multiple tissues and at various developmental stages, suggesting they may have conserved and diverse functions (Figure 9 and Figure 12).

Gene duplication events are major drivers of gene family expansion. In pineapple, nine segmental and one tandem duplication event were identified, suggesting that segmental duplication played a predominant role in the evolution of the *AcLEA* family, consistent with findings in tomato [67], cotton [15], and poplar [63] (Figure 5 and Table S5). Notably, six of the nine segmentally duplicated gene pairs belonged to the LEA_2 subfamily. This is consistent with the observed LEA_2 dominance and may underlie functional expansion in response to cold stress. All duplicated gene pairs were derived from the same subfamily: *AcLEA31/AcLEA11*, *AcLEA17/AcLEA21*, *AcLEA6/AcLEA8*, and *AcLEA35/AcLEA11* from LEA_2; *AcLEA16/AcLEA2* from LEA_5; *AcLEA1/AcLEA24* from LEA_6; and *AcLEA13/AcLEA4* from SMP. The only tandem duplication pair, *AcLEA26/AcLEA27*, belonged to the Dehydrin subfamily. Expression profiling revealed that most duplicated gene pairs exhibited similar tissue-specific and/or developmental-stage-specific redundancy or co-regulation (Figure 9 and Figure 12). For instance, *AcLEA31/AcLEA11* were clustered in Block A and predominantly expressed during sepal development and the early stages of petal development. *AcLEA5/AcLEA9*, *AcLEA1/AcLEA24*, and *AcLEA26/AcLEA27*, all grouped in Block B, were mainly expressed in late-stage stamens, the late stage of gynoecium development (Gy6), and leaves. *AcLEA35/AcLEA29* clustered in Block D and showed high expression in sepals, late-stage petals, and developing fruits. *AcLEA17/AcLEA21* were grouped in Block E and highly expressed in late-stage petals. These consistent expression patterns aligned with the structural analysis, which showed that proteins within the same subfamily shared similar motif compositions and conserved domain features. However, two duplicated gene pairs displayed divergent expression patterns, suggesting potential functional diversification. A notable example is *AcLEA35* and *AcLEA11*: *AcLEA35* was clustered in Block D and preferentially expressed in sepals, petals, and fruit, while *AcLEA11* grouped in Block A and showed different expression specificity. Structural analysis revealed that *AcLEA11* expression patterns, implying potential functional contained an additional motif (motif 9) compared to *AcLEA35* (Figure 2). This structural difference may contribute to their functional divergence. Furthermore, Only the *AcLEA35*/*AcLEA11* duplicated gene pair exhibited a Ka/Ks ratio greater than 1, indicating positive selection (Table S5). This signal of positive selection may reflect ongoing functional divergence shaped by selective pressures associated with pineapple’s adaptation to environmental challenges. Additionally, another duplicated gene pair, *AcLEA6/AcLEA8*, which exhibited distinct expression patterns, displayed a similar phenomenon: *AcLEA8* also contained an additional motif 9 compared to *AcLEA6*. These findings suggest that the presence of the additional motif 9 may confer novel regulatory or protein interaction capabilities, enabling distinct functional roles.

Previous studies have shown that LEA proteins play crucial roles in plant responses to abiotic stresses. *Cis*-element analysis revealed that the promoters of *AcLEA* genes were enriched with stress-responsive elements such as ABRE (ABA-responsive), STRE and LTR (low-temperature-responsive), and CGTCA-motif (MeJA-responsive) (Figure 4 and Table S4). This widespread presence suggests their involvement in multiple stress response pathways. Similar observations were made in *Arabidopsis*, where 82% and 69% of *LEA* promoters contained ABRE and LTR elements, respectively, and most genes were significantly induced by ABA and low temperatures [9]. In addition, transcriptome and qRT-PCR analyses showed that the expression of many *AcLEA* genes was markedly induced under cold stress, especially in the LEA_2 subfamily (*AcLEA32, AcLEA7, AcLEA9, AcLEA30, AcLEA29*), along with members from LEA_1 (*AcLEA33*) and Dehydrin (*AcLEA18*) (Figure 10, Figure 11 and Figure 12). Their expression levels peaked under low temperatures and declined upon stress removal, suggesting dynamic regulation in response to cold stress. Previous studies have shown that LEA_2 subfamily genes are frequently involved in cold stress responses across various plant species. For example, *PtLEA10* and *PtLEA22* not only have seasonal rhythms consistent with cold acclimatization, but are also able to respond to low temperatures [45]. In addition, genes from other LEA subfamilies have also been reported to participate in cold stress responses. For instance, *BnaA.LEA6.a* in *Brassica napus* was identified as strongly cold-responsive and functionally validated to confer freezing tolerance in transgenic lines [66]. Gene structural analysis further revealed that the *AcLEA* family had relatively simple exon-intron structures, with 11 members being intronless. The intron-free genes were mainly distributed in the LEA_2 and LEA_6 subfamilies (Figure 2). In poplar [63], peanut [69], pepper [64], and flax (*Linum usitatissimum*) [70], intron-free *LEA* genes were also distributed within these two subfamilies. qRT-PCR analysis showed that *AcLEA10* and *AcLEA17* without introns were significantly up-regulated after 4 h of cold stress (Figure 11 and Figure 12). Such structural compactness is thought to facilitate rapid gene transcription and energy-efficient responses to environmental cues. Furthermore, cold response patterns differed between the cold-tolerant cultivar BL and the cold-sensitive cultivar NN. Although many *AcLEA* genes were induced in both genotypes, expression in BL was typically faster and stronger. For instance, *AcLEA32* and *AcLEA33* were markedly upregulated in BL but showed limited induction in NN. While *AcLEA7* and *AcLEA9* were both induced earlier with up-regulated expression levels in BL compared to NN, indicating a faster cold response in BL. Such differences suggest that the enhanced expression of specific *AcLEA* genes in BL may underlie its greater cold tolerance and contribute to varietal differences in stress resilience.

LEA proteins have been widely recognized for their roles in abiotic stress tolerance; however, accumulating evidence suggests that they also participate in diverse developmental processes beyond stress adaptation [47,64,66]. In this study, multiple lines of evidence supported the multifunctionality of *AcLEA* genes in pineapple. Subcellular localization predictions indicated that *AcLEA* proteins were distributed across various cellular compartments, including the nucleus, cytoplasm, chloroplasts, mitochondria, plasma membrane, vacuole, endoplasmic reticulum, and peroxisome (Table S1). Consistent with previous studies, such broad distribution patterns may provide protection for different compartments in cells [71]. For instance, LEA proteins such as pea LEA3 have been experimentally shown to localize in mitochondria and protect mitochondrial enzymes like thiocyanate and fumarase from dehydration-induced inactivation [72]. Transcription factor prediction revealed that *AcLEA* genes were potentially regulated by a wide array of transcription factor families, including several known to be involved in abiotic stress responses, such as AP2, CAMTA, NAC, Dof, and MYB (Figure 7 and Table S7). These findings align with previous studies; for instance, *LEA* genes in *Arabidopsis* are regulated by ERF, NAC, and WRKY transcription factors [73], and rice *OsLEA3-1* is regulated by drought-responsive transcription factors such as *OsNAC3*, *OsNAC5*, *ONAC045*, and *OsbZIP23* [74,75,76,77]. In plants, the miRNAs found are negative regulators of target gene, that is, inhibiting the expression of target genes, mainly by transcript cleavage and translation repression [78]. A total of 143 miRNAs were predicted to target 25 *AcLEA* genes in the miRNA network, of which *AcLEA18* (LEA_1) had the most miRNA targeting sites (53), followed by *AcLEA33* (LEA_1) with 13. Transcriptome data and qRT-PCR results showed that the expression levels of these genes were significantly increased after cold treatment. These results indicate that miRNAs inhibit the expression of *AcLEA* genes through transcript cleavage. In addition, *AcLEA* genes were also predicted to be regulated by several development-related transcription factor families, such as MIKC_MADS, GRAS, and bZIP, suggesting their potential involvement in plant growth and organ development. Promoter *cis*-regulatory element analysis further supported this dual role, showing enrichment of not only stress-related elements (e.g., ABRE, STRE, LTR) but also elements involved in plant growth and development (Figure 4). Correspondingly, tissue-specific expression profiles indicated that many *AcLEA* genes were preferentially expressed in floral organs and developing fruit (Figure 9 and Figure 12). This suggests their involvement in reproductive organ development and functional differentiation among tissues. Similar findings have been reported in other species, such as tomato, where *LEA* genes show dynamic expression patterns across various organs and developmental stages [67]. Moreover, LEA proteins have been reported to accumulate during specific growth phases characterized by limited water availability, such as seed and pollen maturation, or particular stages of root and shoot growth [34,35,79]. Protein structure modeling demonstrated that *AcLEA* proteins adopted varied conformations, consistent with their classification into multiple subfamilies (Figure 3). The presence of intrinsic disorder and α-helical structures, both hallmarks of LEA proteins, supports their functional flexibility in stabilizing proteins, membranes, and cellular structures under stress. This structural versatility may also enable their participation in diverse biological processes. Taken together, these results suggest that *AcLEA* genes serve multifaceted roles in pineapple, not only in conferring tolerance to abiotic stresses but also in supporting growth and developmental processes. This dual functionality highlights their value as candidate genes for breeding programs aimed at improving both stress resilience and developmental traits in pineapple.

## 5. Conclusions

In this study, 37 *AcLEA* genes were identified and classified into six subfamilies. Most AcLEA proteins were predicted to be hydrophilic, thermostable, and intrinsically disordered. Segmental duplication was identified as the main driver of gene family expansion. Most duplicated gene pairs exhibited similar gene structures, motif compositions, and expression patterns; however, some showed evidence of divergence. Promoter *cis*-regulatory element, transcription factor, and miRNA network analyses revealed that *AcLEA* genes were potentially involved in responses to multiple abiotic stresses, particularly cold stress, as well as plant growth and developmental regulation. Expression profiling across different tissues and developmental stages showed spatial and temporal specificity, with several *AcLEA* genes such as *AcLEA18* and *AcLEA29* highly expressed across diverse floral organs and fruit. Transcriptome and qRT-PCR analyses showed that many *AcLEA* genes, such as *AcLEA32*, *AcLEA7*, *AcLEA9*, *AcLEA30*, *AcLEA29*, *AcLEA33*, and *AcLEA18*, were strongly induced by cold stress and downregulated upon stress relief, suggesting their potential roles in cold adaptation. Some of these genes such as *AcLEA32* and *AcLEA33* responded more quickly and strongly in the cold-tolerant cultivar BL compared to the cold-sensitive cultivar NN. These genotype-specific differences in expression responsiveness may underlie the distinct cold tolerance observed between cultivars. This work provides valuable insights into the functional diversity of *AcLEA* genes and lays a foundation for their potential use in molecular breeding aimed at improving cold tolerance in pineapple and other tropical crops.

## Data Availability

The data presented in this study are available in the article, Supplementary Materials and online repositories. The pineapple genomic information was retrieved from the Phytozome database (version 3; https://phytozome-next.jgi.doe.gov/info/Acomosus_v3, accessed on 20 May 2025). RNA-seq datasets of pineapple floral organs were obtained from the European Nucleotide Archive (ENA) database under accession number PRJEB38680. Transcriptomic data covering different stages of fruit development were downloaded from the iPlant Collaborative Data Store (https://de.iplantcollaborative.org/de/?type=data&folder=/iplant/home/cmwai/coge_data/Pineapple_tissue_RNAseq, accessed on 21 May 2025). RNA-seq data from pineapple subjected to cold treatment were retrieved from the China National GeneBank DataBase (CNGBdb) database under accession number CNP0008093.

## Supplementary Materials

The following supporting information can be downloaded at: https://www.mdpi.com/article/10.3390/biology14121655/s1, Figure S1: The secondary structure of AcLEA proteins; Table S1: Detailed information on 37 LEA genes in pineapple; Table S2: Gene IDs and nomenclature of LEA gene family in pineapple and Arabidopsis; Table S3: Prediction information and homologous modeling evaluation of pineapple LEA protein secondary structure; Table S4: Prediction of cis regulatory elements in the promoter region of pineapple LEA gene; Table S5: The Ka/Ks ratio of repeated LEA gene pairs in pineapple; Table S6: Statistical analysis of collinear gene pairs between pineapple and four other representative species; Table S7: Predictive transcription factors involved in regulating all LEA genes in pineapple; Table S8: Detailed information on the interaction between predicted miRNAs in pineapple and their targeted LEA genes; Table S9: Primers for qRT-PCR analysis of LEA gene in pineapple.
